# Neuromodulation impact on nonlinear firing behavior of a reduced model motoneuron with the active dendrite

**DOI:** 10.3389/fncom.2014.00110

**Published:** 2014-09-09

**Authors:** Hojeong Kim, C. J. Heckman

**Affiliations:** ^1^Division of Robotics Research, Daegu Gyeongbuk Institute of Science and TechnologyDaegu, South Korea; ^2^Department of Physiology, Northwestern UniversityChicago, IL, USA; ^3^Department of Physical Medicine and Rehabilitation, Northwestern UniversityChicago, IL, USA; ^4^Department of Physical Therapy and Human Movement Science, Northwestern UniversityChicago, IL, USA

**Keywords:** neuromodulation, motoneurons, nonlinear firing, persistent inward current, reduced modeling, computer simulation

## Abstract

Neuromodulatory inputs from brainstem systems modulate the normal function of spinal motoneurons by altering the activation properties of persistent inward currents (PICs) in their dendrites. However, the effect of the PIC on firing outputs also depends on its location in the dendritic tree. To investigate the interaction between PIC neuromodulation and PIC location dependence, we used a two-compartment model that was biologically realistic in that it retains directional and frequency-dependent electrical coupling between the soma and the dendrites, as seen in multi-compartment models based on full anatomical reconstructions of motoneurons. Our two-compartment approach allowed us to systematically vary the coupling parameters between the soma and the dendrite to accurately reproduce the effect of location of the dendritic PIC on the generation of nonlinear (hysteretic) motoneuron firing patterns. Our results show that as a single parameter value for PIC activation was either increased or decreased by 20% from its default value, the solution space of the coupling parameter values for nonlinear firing outputs was drastically reduced by approximately 80%. As a result, the model tended to fire only in a linear mode at the majority of dendritic PIC sites. The same results were obtained when all parameters for the PIC activation simultaneously changed only by approximately ±10%. Our results suggest the democratization effect of neuromodulation: the neuromodulation by the brainstem systems may play a role in switching the motoneurons with PICs at different dendritic locations to a similar mode of firing by reducing the effect of the dendritic location of PICs on the firing behavior.

## Introduction

Spinal motoneurons have large, highly branched dendrites and voltage-gated ion channels that generate strong persistent inward currents (PICs) (Schwindt and Crill, 1980). Over the past 30 years, the impact of PICs on the firing output of the motoneurons has been extensively investigated in various species, including turtles (Hounsgaard and Kiehn, 1985, 1989), rats (Bennett et al., 2001; Li and Bennett, 2003), mice (Carlin et al., 2000; Meehan et al., 2010) and cats (Lee and Heckman, 1998, 1999). There has been a consensus in the motoneuron physiology community that in the presence of monoamines (i.e., norepinephrine and serotonin), the activation of the L-type Ca^2+^ PIC channels is facilitated, producing a long-lasting membrane depolarization (i.e., plateau potential) (reviewed in Powers and Binder, 2001; Heckman et al., 2008). The spatiotemporal interaction between the spike-generating channels at the soma and the plateau-generating PIC channels at the dendrites, may be the mechanism underlying the nonlinear (e.g., bistable) firing of the motoneurons.

The firing patterns of the motoneurons have been characterized experimentally using slowly rising and falling current stimulation to the soma. Four types of firing have been identified during this triangular current clamp, characterized by the relationship between their frequency and current intensity (F-I) (Bennett et al., 2001; Button et al., 2006; Cotel et al., 2009): Type I, a linearly overlapped F-I curve; Type II, lower firing rates during the falling phase than during the rising phase of the stimulation, showing clockwise hysteresis for the F-I curve; Type III, a linearly overlapped F-I curve with sustained firing on the descending phase of the stimulation below the threshold for spike initiation; and Type IV, higher firing rates during the falling phase than during the rising phase of the stimulation, showing counterclockwise hysteresis for the F-I curve, with sustained firing behavior. Type IV firing has also been referred to as “fully bistable” firing, whereas when the plateau potential is deactivated at a higher current level during the descending stimulation phase than the threshold for spike initiation, Type IV firing has been called “partially bistable” (Lee and Heckman, 1998). In particular, the Type III and IV firing patterns have been associated with the activation of plateau potentials mediated by the PIC channels in the dendrites. In the present study, the terms “fully hysteretic” and “partially hysteretic” were used for Type IV firing instead of “fully bistable” and “partially bistable” due to their compound meaning from a dynamic systems perspective.

The location of the PIC channels is a crucial factor for generating nonlinear (i.e., fully hysteretic Type IV) firing in motoneurons. Many experimental and computational studies have suggested that the PIC channels must not be uniformly distributed but rather clustered near the middle (i.e., 300–600 μm from the soma) of the dendrites for fully hysteretic, Type IV firing patterns (Hounsgaard and Kiehn, 1993; Carlin et al., 2000, 2009; Elbasiouny et al., 2005; Ballou et al., 2006; Bui et al., 2006). In our recent computational studies (Kim and Jones, 2011, 2012; we have further demonstrated 1) that the types of firing patterns (I–IV) can be generated by simply changing the dendritic location of a constant amplitude PIC and 2) that these location-dependent effects of firing depend on the attenuation of voltage along the dendrites and that both the dendrite-to-soma and soma-to-dendrite attenuation behaviors are important.

Another factor that may play a critical role in determining the firing output is the neuromodulatory inputs (in particular monoamines) from the brainstem to the motoneurons. The primary effect of neuromodulation is a profound facilitation of PIC activation, presumably via G-protein-mediated signaling pathways (Hille, 2001), which leads to an increase in the intrinsic excitability of the motoneuron dendrites. However, it remains unclear how the interplay between the neuromodulation effect and the PIC location influences the firing dynamics of the motoneurons. In this study, we used our recently developed reduced modeling approach, which allowed us to explicitly manipulate these two key factors determining the nonlinear dynamics of the motoneurons: *location* and *neuromodulation effect on the PIC*.

## Materials and methods

### The conductance-based, reduced neuron model

The structure of the neuron model used in this study is similar to the conventional two-compartment model, which consists of the somatic and dendritic compartments, coupled by electrotonic coupling. Each compartment can be characterized separately by its specific membrane conductance (*G_m,S_* and *G_m,D_*) and capacitance (*C_m,S_* and *C_m,D_*), and connected together via a coupling conductance (*G_C_*). The major difference from the conventional reduced modeling approach is that five passive parameters (*G_m,S_*, *G_m,D_*, *C_m,S_*, *C_m,D_*, and *G_C_*) of our reduced model can be analytically determined to retain five system properties obtained from the anatomically reconstructed motoneuron: input resistance (*R_N_*), membrane time constant (τ_*m*_) and three voltage attenuation (VA) factors between the soma and the dendrites:

- *VA^DC^_SD_* is the ratio (*V_dendrite_/V_soma_*) of voltage at the dendrites to voltage at the soma for DC input at the soma.
- *VA^AC^_SD_* is the ratio (*V_dendrite_/V_soma_*) of voltage at the dendrites to voltage at the soma for AC input at the soma.
- *VA^DC^_DS_* is the ratio (*V_soma_/V_dendrite_*) of voltage at the dendrites to voltage at the soma for DC input at the dendrites.

The five system properties are related analytically to the five cable parameters of the reduced model as follows,

$$\begin{array}{l} G_{m,S}=\frac{1-VA_{DS}^{DC}}{r_{N}(1-VA_{SD}^{DC}VA_{DS}^{DC})}\\ \;G_{m,D}=\frac{pVA_{DS}^{DC}(1-VA_{SD}^{DC})}{\left(1-p)r_{N}VA_{SD}^{DC}(1-VA_{SD}^{DC}VA_{DS}^{DC}\right)}\\ \;G_{C}=\frac{pVA_{DS}^{DC}}{r_{N}(1-VA_{SD}^{DC}VA_{DS}^{DC})}\\ \;C_{m,D}=\frac{1}{\omega (1-p)}\sqrt{\frac{G_{C}^{2}}{{\left(VA_{SD}^{AC}\right)}^{2}}-{\left\{G_{C}+G_{m,D}(1-p)\right\}}^{2}}\\ C_{m,S}=\frac{\begin{array}{c} \tau_{m}\{p\left(1-p\right)\tau_{m}G_{m,S}G_{m,D}+pG_{m,S}\left(\tau_{m}G_{C}-C_{m,D}\right)\\ \;+p^{2}G_{m,S}C_{m,D}+\left(1-p\right)\left(\tau_{m}G_{C}G_{m,D}-G_{C}C_{m,D}\right)\} \end{array}}{\;p\left\{\left(1-p\right)\left(\tau_{m}G_{m,D}-C_{m,D}\right)+\tau_{m}G_{C}\right\}} \end{array}$$

where *r_N_* is the input resistance (*R_N_*) normalized with the surface area of the somatic compartment; ω is the maximum frequency component in an action potential; *p* is the ratio of somatic to total surface area of the reduced model. In the present study, the values of *r_N_* (0.198), τ_*m*_ (10.4), *p* (0.168). and ω (2π × 250) were calculated based on the experimental data and set to be constant for simulations (Kim and Jones, 2012).

The calculation of the system properties from the anatomical model and the derivation of inverse equations for the cable parameters of the reduced model were fully presented in our previous studies (Kim et al., 2009; Kim and Jones, 2012). The Morris-Lecar type of membrane excitability was added to the soma to generate the spikes mediated by lumped inward current (represents fast Na^+^) and outward current (represents delayed rectified K^+^) and to the dendrite to produce the plateau potentials mediated by the L-type Ca^2+^ and K^+^ currents.

The somatic (*V_S_*) and dendritic (*V_D_*) membrane potential responses to the somatically injected current (*I_S_*) were governed by the following current-balance equation at the somatic compartment,

(1) $$\begin{array}{l} C_{m,S}\overset{•}{V_{S}}=-G_{m,S}(V_{S}-E_{Leak})-\frac{G_{C}}{p}(V_{S}-V_{D})-G_{Na}\\ \;m_{S\infty }(V_{S}-E_{Na})-G_{K,S}n_{S}(V_{S}-E_{K})+I_{S} \end{array}$$

(2) $$m_{S\infty }(V_{S})=0.5\text{​}\left(1+\tanh\frac{V_{S}+0.01}{0.15}\right)$$

(3) $$\begin{array}{l} \overset{•}{n_{S}}=0.2\text{​}\left(\frac{n_{S\infty }(V_{S})-n_{S}}{\tau_{S}(V_{S})}\right)\text{ where}\\ n_{S\infty }(V_{S})=0.5\text{​}\left(1+\tanh\frac{V_{S}+0.04}{0.1}\right),\\ \tau_{S}(V_{S})={\left(\cosh\frac{V_{S}+0.04}{0.1}\right)}^{-1} \end{array}$$

and the dendritic compartment,

(4) $$\begin{array}{l} C_{m,D}\overset{•}{V_{D}}=-G_{m,D}(V_{D}-E_{Leak})-\frac{G_{C}}{1-p}(V_{D}-V_{S})\\ \;-\;G_{Ca}m_{D}(V_{D}-E_{Ca})-G_{K}{,}_{D}{{}_{n}}_{D}(V_{D}-E_{K}) \end{array}$$

(5) $$\begin{array}{l} \overset{\text{​}•}{m_{D}}=0.2\text{​}\left(\frac{m_{D\infty }(V_{D})-m_{D}}{\tau_{mD}(V_{D})}\right)\text{ where}\\ m_{D\infty }(V_{D})=0.5\text{​}\left(1+\tanh\frac{V_{D}-V_{1D}}{V_{2D}}\right),\\ \tau_{mD}(V_{D})={\left(\cosh\frac{V_{D}-0.07}{0.1}\right)}^{-1} \end{array}$$

(6) $$\begin{array}{l} \overset{•}{n_{D}}=0.2\text{​}(\frac{n_{D\infty }(V_{D})-n_{D}}{\tau_{nD}(V_{D})})\;where\\ n_{D\infty }(V_{D})=0.5\text{​}(1+\tanh\frac{V_{D}}{0.1}),\\ \tau_{nD}(V_{D})={\left(\cosh\frac{V_{D}}{0.1}\right)}^{-1} \end{array}$$

where the subscripts *S* and *D* indicate the soma and dendrite; The initial values for maximum conductances of active currents were *G_Na_* = 11.0, *G_K,S_* = 14.0, *G_Ca_* = 0.89, and *G_K,D_* = 0.44; *m* and *n* are activation functions for inward and outward active currents; The reversal potentials for individual ions were *E_Na_* = 1.0, *E_K_* = −0.7, *E_Ca_* = 1.0, and *E_Leak_* = −0.5; The initial values for the half-activation voltage and one over the slope of the activation curve for the voltage-gated Ca^2+^ current at the dendrite were current at the dendrite were *V_1D_* = 0.07 and *V_2D_* = 0.1. All parameter values were adopted from our previous model that produced fully hysteretic, Type IV firing. The numbers are dimensionless, unless otherwise stated (see the dimensionless analysis in Rinzel and Ermentrout, 1998).

### Simulations

**Figure 1:**
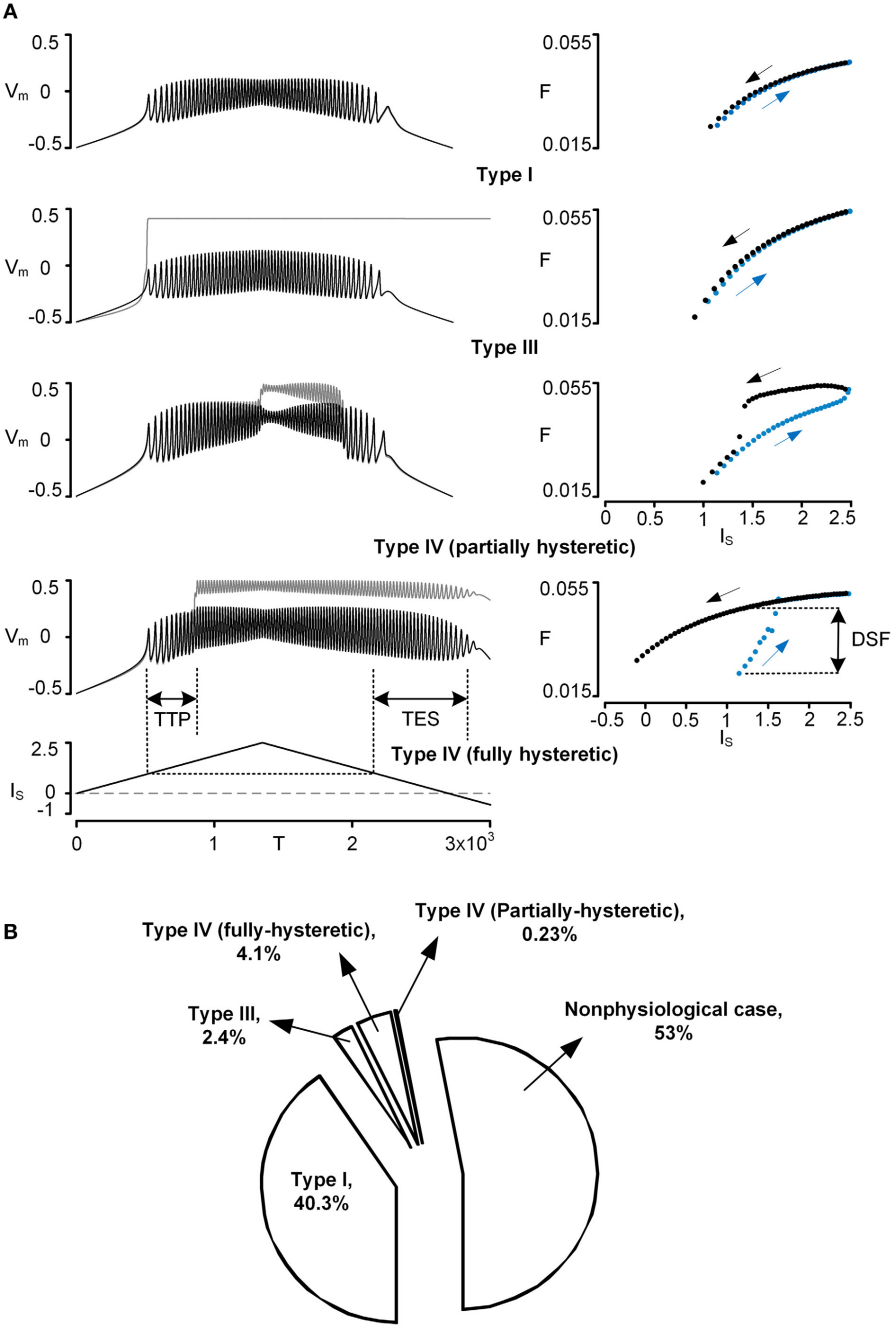
**Physiological firing patterns of a reduced motoneuron model**. Firing patterns were simulated while systematically varying three VA factors over a whole range of values (0–1). **(A)** Four examples of physiological firing types (Types I–IV): time courses of voltage responses (*V_m_*) of the soma (black solid lines) and the dendrite (gray solid lines) to the triangular current stimulation (*I_S_*) to the soma (left column) and the corresponding frequency-current (F–*I_S_*) curves (blue and black dots for ascending and descending phase of the current stimulation, right column). To automatically process the detection of firing types during simulations, the three characteristic indexes (TTP, TES, DSF) were defined based on both *V_m_*–*I_S_* and F–*I_S_* responses; TTP, TES, and DSF indicate time to onset of plateau potential, time to end of somatic spiking, and difference in spiking frequency, respectively. For instance, Type I firing was detected when all characteristic indexes were zero, whereas Type IV firing (fully hysteretic) was detected when all characteristic indexes were positive. Note that *V_m_*, *I_S_*, T, and F are dimensionless. The values of VA factors (*VA^DC^_SD_*, *VA^DC^_DS_*, *VA^AC^_SD_*) and cable parameters (*G_m,S_*, *G_m,D_*, *G_C_*, *C_m,S_*, *C_m,D_*) for the reduced models are (0.97, 0.63, 0.84) and (4.805, 0.051, 1.375, 49.499, 0.626) for Type I firing, (0.65, 0.003, 0.08) and (5.045, 0.002, 0.003, 52.425, 0.024) for Type III firing, (0.96, 0.57, 0.81) and (4.796, 0.054, 1.068, 49.952, 0.542) for Type IV (partially hysteretic) firing, and (0.94, 0.38, 0.69) and (4.871, 0.039, 0.502, 50.772, 0.378) for Type IV (fully hysteretic) firing. **(B)** Pie chart: the size of the individual pieces corresponds to the number of points in the VA space at which the model produces the specific firing types. Nonphysiological case indicates when there is no real solution for the model parameter values or no spiking at all (see Kim and Jones, 2012, for the full description).

The firing behavior of the reduced model was simulated by applying the triangular current stimulation (peak of 2.5 with a duration of 3000) to the soma. The simulation was performed while varying individual VA factors independently over a full range (0~1). To facilitate the process of identifying the firing types (i.e., Type I–IV) during simulations, three characteristic indices (CIs) were defined based on spike trains and firing frequency (see the bottom panel in Figure 1A for graphical explanation):

- Time To onset of Plateau potential (TTP): this index measures the latency between the first somatic action potential and the onset of the dendritic plateau potential. If this value is positive, the onset of the plateau potential follows the first somatic spike. If the value is negative, the plateau potential precedes somatic spiking.
- Time to End of somatic Spiking (TES): this index measures the duration of spiking during the downward phase of current stimulation relative to the current threshold from the upward phase. If this value is positive, somatic spiking persists past the spiking threshold on the upward phase. If the value is negative, spiking stops before reaching the threshold determined on the upward phase of stimulation.
- Difference in Spiking Frequency (DSF): this index measures the difference in instantaneous spiking frequency at the current threshold determined on the upward phase of stimulation. If this value is positive, the firing frequency is greater on the downward phase and indicates counter-clockwise frequency hysteresis. If this value is negative, spiking frequency on the downward phase is less or repetitive spiking has ceased.

The signs of individual CIs were operationally evaluated to determine the firing type while varying individual VA factors. For instance, the Type I–III firing behavior could be detected when [TTP = 0, DSF = 0, TES = 0], [TTP = 0, DSF < 0, TES = 0] and [TTP = 0, DSF = 0, TES > 0], respectively. Notably, the Type IV firing of particular interest displayed all positive CIs.

**Figure 2:**
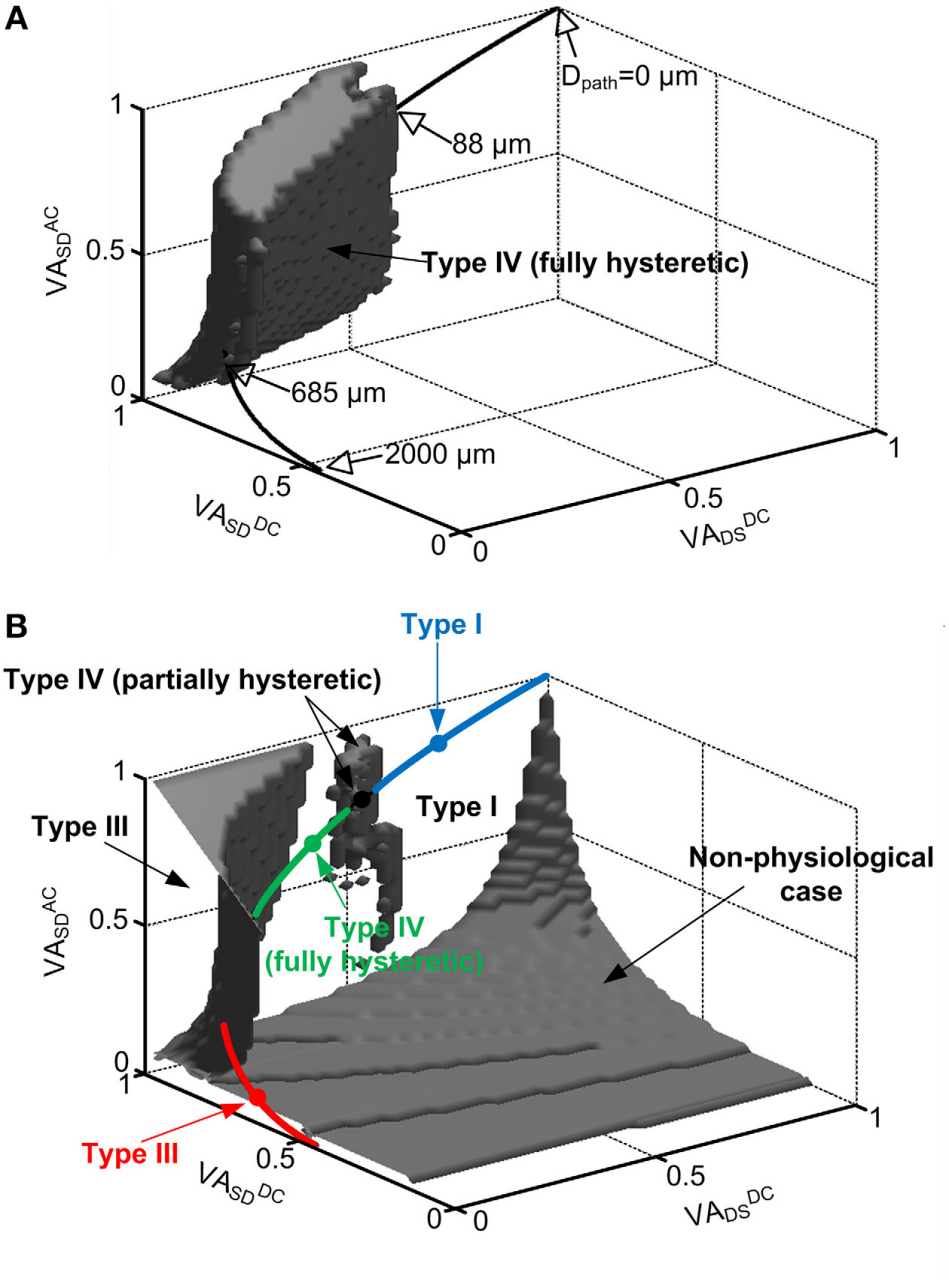
**Location dependent firing patterns of a reduced motoneuron model**. The gray volumes in the 3D VA space indicate the values of the VA factors with which the reduced model generated: fully hysteretic Type IV firing **(A)** showing all positive characteristic indexes, Type III firing in the upper-left corner **(B)**, partially hysteretic Type IV firing in the upper-middle region **(B)**, and nonphysiological case in the lower-right corner **(B)** where cable parameters are negative or somatic capacitance to produce system time constant does not exist. The rest space outside the gray volumes represents the VA values for Type I firing. In both **(A,B)**, the physiological VA factors obtained at all distances (D_path_) from the soma of the anatomical motoneuron models were mapped on the same VA space by statistically plotting their mean values (thick solid lines). The open arrows indicate the physical locations (D_path_) of the CaPIC channel in the 3D VA space, whereas the close arrows indicate the firing types that the reduced model can produce with the physiological values of the VA factors. The four closed circles (blue, black, green, red) on the line for the mean of physiological VA data indicate the points where the reduced model produces Type I, IV (partially hysteretic), IV (fully hysteretic) and III firing shown in Figure 1A. The blue, green and red sold line show the region of physiological VA factors for Type I, IV (fully hysteretic) and III firing, respectively. Note that in the physiological case, all three VA factors are 1 at the soma (D_path_ = 0) and decrease approaching the dendritic terminals (D_path_ = 2000 μm).

All of the sets of VA factor values that produced the same firing type with the reduced model were plotted as points in the three-dimensional (3D) VA space defined as x = *VA^DC^_DS_*, y = *VA^DS^_SD_*, and z = *VA^AC^_SD_*. To map the VA space on the physical locations of the dendritic trees, five type-identified motoneurons were fully reconstructed in the NEURON environment. The physiological data for each VA factor were measured as a function of the distance (D_path_) from the soma of the anatomical motoneuron models and fitted to a single exponential function [i.e., exp(−D_path_/η)] to represent the spatial profile of the voltage attenuation with the single parameter (η). Using the fitting functions (η = {2680.6, 3059.5, 2758, 1941, 2145.8} for *VA^DC^_SD_*, {224.2, 144.7, 119.5, 143.9, 190.8} for *VA^DC^_DS_*, and {420.1, 437.1, 402.3, 373.1, 464.7} for *VA^AC^_SD_*), the mean for individual VA factors (*VA^DC^_SD_*, *VA^DC^_DS_*, *VA^AC^_SD_*) was investigated as a function of D_path_ for the VA data. The physiological VA data were represented by plotting their mean values in the 3D VA space with assumption of normal distribution of physiological VA values at a specific distance from the soma (see thick solid lines, Figures 2, **4**). All details about the anatomical model reconstruction and VA analysis were fully addressed in our previous studies (Kim et al., 2009; Kim and Jones, 2012). The dendritic locations of PIC channels for generating the Type IV (fully hysteretic) firing were estimated by superimposing the physiological VA data associated with the distance on the solution VA space for the Type IV firing in the 3D VA space.

The facilitating effect of monoaminergic neuromodulation on the activation of the PIC in the dendrites was simulated by varying either individual or all three activation parameters [i.e., *G_Ca_ V_1D_*, *V_2D_*, and in Equations (4) and (5)] of CaPIC channels in the dendrite from −100 to 100% of their standard values. Given the activation parameter values for the CaPIC channel, all sets of VA factor values that produced positive values for the three CIs were plotted in the VA space. The variation in the dendritic locations of the PIC channels for the Type IV firing under neuromodulatory effects was evaluated recognizing the intersection area between the theoretical VA solution and the physiological VA data in the VA space.

## Results

### PIC location dependency of physiological firing patterns in the reduced model

Firing patterns of the reduced model were simulated with triangular current stimulation to the soma while varying individual voltage attenuation (VA) factors (i.e., *VA^DC^_SD_*, *VA^DC^_DS_*, and *VA^AC^_SD_*). We first evaluated the capability of the reduced model to produce physiological firing patterns that have been observed experimentally from spinal motoneurons. Based on the results from the VA analysis, the locations of the persistent inward current (PIC) for individual firing patterns were then estimated as a function of the path length from the soma by comparing the VA factor values with those measured from the anatomically reconstructed motoneuron models.

#### Model capability for generating physiological firing patterns

Four types of five physiologically observed firing patterns [i.e., Type I–III and IV (fully and partially hysteretic) except Type II] could be explicitly reproduced by the reduced model during the computer simulations (Figure 1A). When the PIC in the dendrite was not activated during the stimulation, the reduced model produced Type I firing with a linearly overlapped F-I relation and without sustained firing in the descending phase of the current stimulation below the rheobase for spike initiation. When the dendritic PIC was activated near the current threshold for spike initiation in the ascending stimulation phase, the reduced model displayed Type III firing, giving a linearly overlapped F-I relation with sustained firing in the descending phase. The Type IV (fully hysteretic) firing, with a counter-clockwise F-I relation with sustained firing, was detected when the onset and offset of the PIC was delayed relative to the spike initiation during the ascending and descending stimulation phase. At a limited range of the voltage attenuation factors near their default values, the PIC during the ascending stimulation phase was deactivated in the descending phase prior to reaching the current threshold for the initiation of action potentials, showing a partially hysteretic Type IV firing pattern. The reduced model rarely produced Type II firing (characterized with the strong clockwise frequency-current relationship without sustained firing) while varying only the voltage attenuation properties between the soma and the dendrite. This result suggests that the location of the PIC channels in the dendrites may not be a main factor of generating Type II firing, supporting the idea that a slow adaptation of firing rate, mediated by active currents at the soma, could be the main mechanism underlying Type II firing (Iglesias et al., 2011).

Overall, the firing patterns produced by the reduced model during the variation of the VA factors were categorized as 40.3% Type I, 2.4% Type III, 4.1% fully hysteretic Type IV, 0.23% partially hysteretic Type IV firing, and 53% nonphysiological (Figure 1B). The distribution of the VA factor values associated with individual firing types in the 3D VA space was graphically presented along with the physiological VA data in the next section. Briefly, Type I firing tended to be generated as the *VA^DC^_SD_* decreased, the *VA^DC^_DS_* increased, and the *VA^AC^_SD_* decreased from their default values. Type III firing was found where the *VA^DC^_SD_* and *VA^AC^_SD_* were much greater than the *VA^DC^_DS_*. Type IV firing, characterized at the default values of three voltage attenuation factors, was much more sensitive to variation of the *VA^DC^_SD_* than the other voltage attenuation factors.

#### Spatial relationship of the firing types

In physiological conditions, the three VA factors characterized between the soma and all individual points of the dendrites are not free parameters to be independently varied but are tightly constrained by the path length from the soma of the cell (Hausser et al., 2000; Bui et al., 2003; Kim and Jones, 2012). For instance, all three VA factors of motoneurons have the same value of 1 at the soma and exponentially decay with increasing distance toward the dendritic terminals depending on the propagation direction and frequency of the signal. To infer the physical locations of the CaPIC channels at which the reduced model generated one of the four firing types, we superimposed the VA factors measured from five anatomically reconstructed motoneurons on the 3D VA space in which each point represented a set of the three VA factor values (Figure 2).

Figure 2 shows that the reduced motoneuron model is capable of producing all four firing types shown in Figure 1 within the physiological range of the VA factors, depending on the location of the PIC. The physiological VA data obtained from the anatomically reconstructed motoneuron models were superimposed over the VA space where the reduced model produces fully hysteretic Type IV firing (Figure 2A). Based on the spatial relationship of the physiological VA factors with the distance from the soma, the fully hysteretic Type IV (i.e., counterclockwise F-I curve with sustained firing) was found to be generated in the intermediate range between 88 μm and 685 μm as reported previously (Elbasiouny et al., 2005; Ballou et al., 2006; Bui et al., 2006). Similarly, the overlap of the physiological VA data on the VA spaces for Type I, III, and IV (partially hysteretic) firing indicated the spatial relationship between the individual firing types and the location of PIC channels in the dendrites (Figure 2B). The partially hysteretic Type IV was evoked at the very limited range of the distance, which was just around the lower bound of the distance range for the fully hysteretic Type IV firing. The Type I (i.e., linearly overlapped F-I curve) firing tended to occur when the PIC channels were placed at proximal sites (<88 μm) to the soma, whereas the Type III (i.e., linearly overlapped F-I curve with sustained firing) was produced at distal sites (>685 μm) to the soma.

In addition, the tendency of the Type I firing (without PIC activation) at the region proximal to the soma and the Type III firing (with PIC activation) at the distal area of the dendrites indicates that the excitability of the dendrites increases as the PIC channel moves toward dendritic terminals from the soma. The increasing excitability of the dendrites with increasing distance may be attributed to the increasing input resistance of the dendrites with the distance (Kim et al., 2009).

The shape of the nonlinear firing pattern was potentially adjustable depending on the PIC location in the dendrites. For instance, the extent of the counterclockwise hysteresis of the Type IV firing was maximized at the proximal distance of 88 μm, whereas it was minimized at the distal distance of 685 μm to the soma (see Figure 6 in Kim and Jones, 2012). All these results emphasize the importance of PIC channel location over the dendritic trees in determining the firing patterns of the motoneurons.

### Neuromodulation effects on PIC location dependence of the firing patterns

Because the molecular mechanisms underlying the neuromodulatory facilitation of PIC activation are still unclear, we first evaluated how individual activation properties of the CaPIC channel in the dendrite influence the PIC characteristics that have been experimentally measured at the soma. Then, the spatial relation of the firing behavior (i.e., Type I–IV) to the PIC locations was reevaluated while varying the activation properties of the CaPIC channel.

#### Dependence of PIC characteristics on CaPIC activation properties

**Figure 3:**
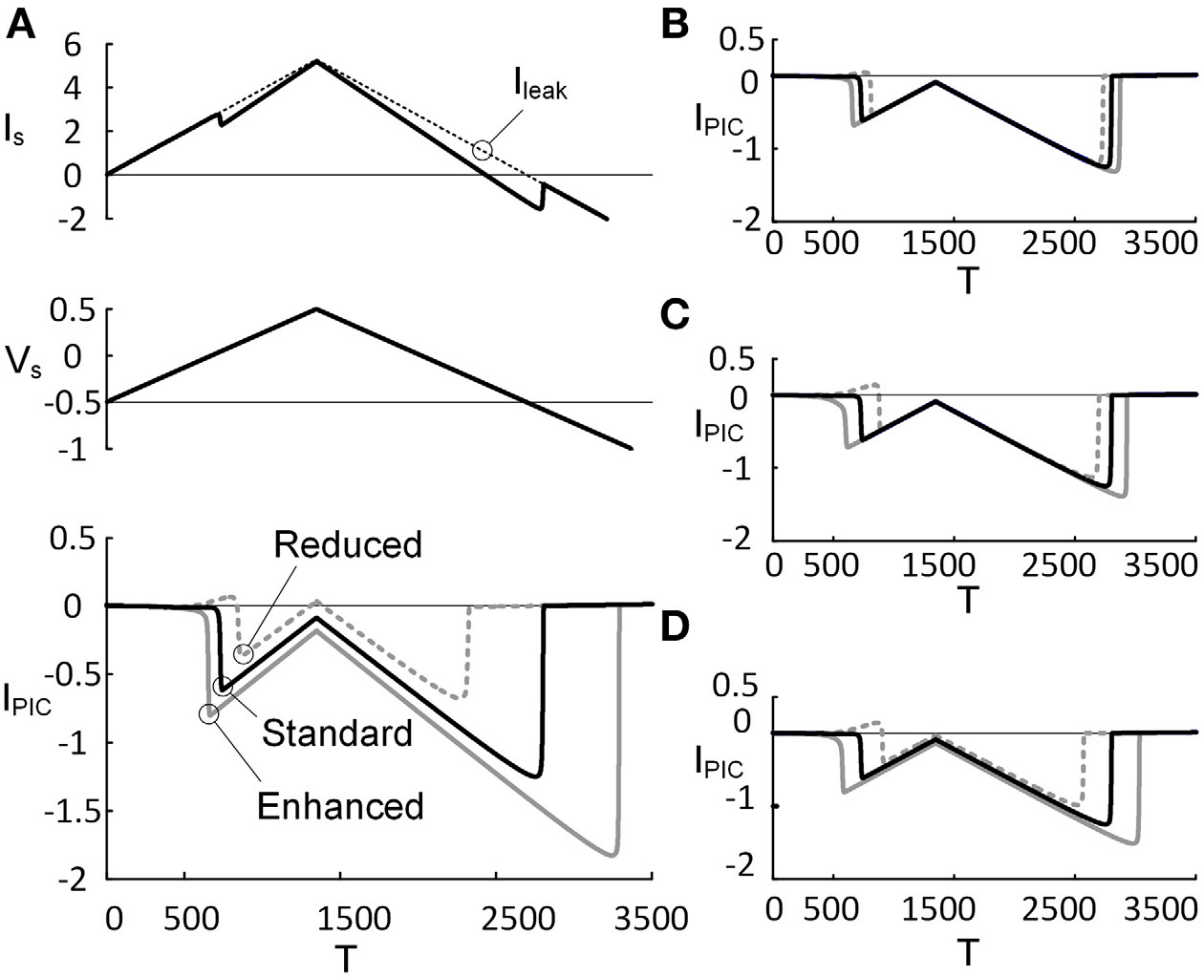
**PIC characteristics depending on PIC activation property**. Three activation parameters (*G_Ca_*, *V_1D_*, and *V_2D_*) underlying the CaPIC activation were varied individually **(A–C)** or all together **(D)** at three different levels (reduced, standard, enhanced) of neuromodulation. **(A)** A triangular voltage clamp (*V_s_*, middle) was applied at the soma and measured the total current (*I_S_*, top) injected to follow up the voltage command (*V_s_*). PIC (I_PIC_, bottom) was calculated by subtracting *I_S_* (solid line, top) from the leak current (I_leak_, dotted line, top). The gray dotted, black solid, and gray solid lines indicate PICs with *G_Ca_* reduced by 20%, default value and increased by 20%, respectively. **(B–D)** The same simulation protocol for A was applied for other parameters: *V_1D_* **(B)**, *V_2D_* **(C)**, and all three parameters **(D)**. Note that at the third case, the individual parameters were varied by 10% in the same direction (inhibiting or facilitating the PIC activation).

The effects of monoaminergic neuromodulation on the PIC (I_PIC_) characterized at the soma were simulated by modulating three activation parameters (i.e., *G_Ca_*, *V_1D_*, and *V_2D_*) that govern the dynamics of the L-type Ca^2+^ channel in the dendrite of the reduced model (Figure 3). The activation parameters were varied by ±20% from their initial values when they were considered individually or by ±10% when all of the parameter values were changed at the same time. The percentage of variation in the activation parameter values was determined to match the physiological variation of the I_PIC_ peak that has been observed experimentally (approximately 31% increased at an enhanced level of neuromodulation and 41% decreased at a reduced level, compared with a moderate level of neuromodulation) (Lee and Heckman, 2000). As expected, an increase in *G_Ca_* (maximum conductance of the L-type Ca^2+^ channel) and *V_2D_* (one over the slope of the activation curve for the L-type Ca^2+^ channel) produced excitatory effects lowering the voltage threshold for the PIC activation and increasing the PIC amplitude (bottom panel of Figures 3A,C), whereas an increase in *V_1D_* (half activation voltage of the activation curve for the L-type Ca^2+^ channel) caused inhibitory effects increasing the voltage threshold for the activation of the dendritic PIC and lowering the PIC amplitude (Figure 3B). Similar results were obtained when these three activation parameters were varied simultaneously to produce the same effect on the activation of the PIC (Figure 3D). Prior to its activation at the reduced level of neuromodulation, a positive I_PIC_ was found in all four cases (Figures 3A–D), indicating an increase in the net outward current due to the decrease in PIC by downgraded neuromodulation. At each level of neuromodulation, no significant difference was found in the onset timing and amplitude of the I_PIC_ during the rising phase of the stimulation between the four manipulations (i.e., change in *G_Ca_*, *V_1D_*, *V_2D_* or all). This result indicates that varying the individual activation parameters of the CaPIC channel in the same inhibitory or excitatory direction has similar effects on the activation of the PIC, when measured at the soma.

#### Influence of neuromodulation on the spatial relationship of the firing types

**Figure 4:**
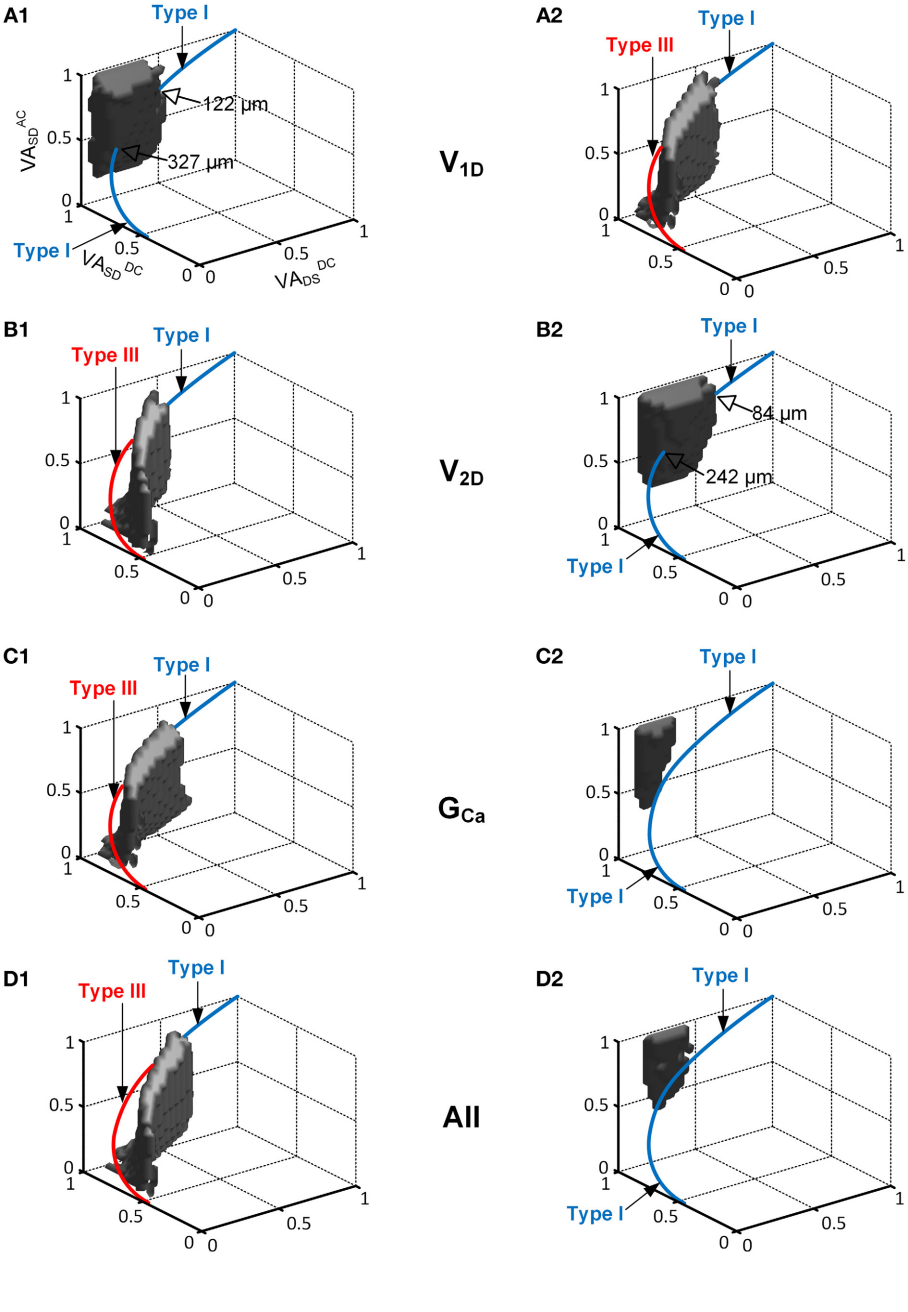
**The interaction of the location and activation properties of PIC channels on the firing patterns**. The same simulation performed in Figure 2 was conducted while varying the individual activation properties (*V_1D_*, *V_2D_*, and *G_Ca_*) of the CaPIC channel. **(A1,B1,C1)** show the simulation results with 20% increments of *V_1D_*, *V_2D_*, and *G_Ca_*, whereas **(A2,B2,C2)** show the results for 20% decreases from their standard values. **(D1,D2)** show the simulation results from a simultaneous change in all of the activation parameter values at the same time, where **(D1)** shows a 10% increase in *V_2D_* and *G_Ca_*, and 10% decrease in *V_1D_* whereas **(D2)** shows the opposite.

Figure 4 demonstrates how varying the PIC activation parameters influences the range of PIC locations over which the reduced motoneuron model can produce distinctive firing types. Overall, the VA space (i.e., gray area in Figure 2), where the reduced model produced the nonlinear firing (i.e., fully hysteretic Type IV) at a moderate level of neuromodulation, dramatically shrank and shifted along the *VA^DC^_SD_* axis in the 3D VA domain, depending on the inhibitory or excitatory effect of the CaPIC parameter variation on the I_PIC_.

When neuromodulation was decreased by decreasing the PIC's *V_1D_* (Figure 4A2) or increasing its *V_2D_* (Figure 4B1) by 20%, the solution space for Type IV firing on the 3D VA plot was significantly reduced and moved downward along the *VA^DC^_SD_* axis. Consequently, the reduced model was only capable of generating Type III firing patterns at most dendritic locations of PIC channels. In contrast, an increase in neuromodulation by increasing *V_1D_* (Figure 4A1) or by decreasing *V_2D_* (Figure 4B2) by 20% caused the solution space for the Type IV firing to move upward along the *VA^DC^_SD_* axis, leading to a significant reduction in the range of the PIC location for Type IV firing of 66 or 74%, relative to the default values. In this case, the reduced model could produce Type I firing only at the distances from the soma outside the reduced range of the PIC location for Type IV firing.

Varying *G_Ca_* showed a larger impact on the size and location of the VA region for the Type IV firing of the reduced model. The CaPIC channel locations for Type IV firing almost disappeared when the value of *G_Ca_* was either increased (Figure 4C1) or decreased (Figure 4C2) by 20%. Consequently, the reduced model could display only two firing modes, Type I or Type III, depending on the modulation level (reduced or enhanced) regardless of PIC channel positions in the dendrites.

Furthermore, when all PIC activation parameters were simultaneously modulated by 10% to either increase (Figure 4D1) or decrease (Figure 4D2) the excitability of the PIC in the dendrites, the location and the size of the Type IV firing space was shifted further away from the physiological voltage attenuation data and decreased more severely than for the case where individual PIC activation parameters were changed independently.

All simulation results suggest that any variation of neuromodulation, either enhancement or reduction, may lead to a significant reduction in the dendritic sites of the CaPIC for Type IV firing, indicating an alleviation of the PIC location effect on the firing behavior.

### The robustness of the reduced model for the nonlinear firing under neuromodulatory control

Having shown the effects of neuromodulation on the nonlinear firing behavior of the reduced model, within the physiological range of the variation in the I_PIC_, we further extended the VA analysis to a broader range of activation parameter values (±100% from default values) to evaluate how robustly the reduced model can produce the nonlinear firing behavior. The robustness of the reduced model for the nonlinear firing was indirectly evaluated by counting the number of points in the VA space where the reduced model produced Type IV firing patterns.

**Figure 5:**
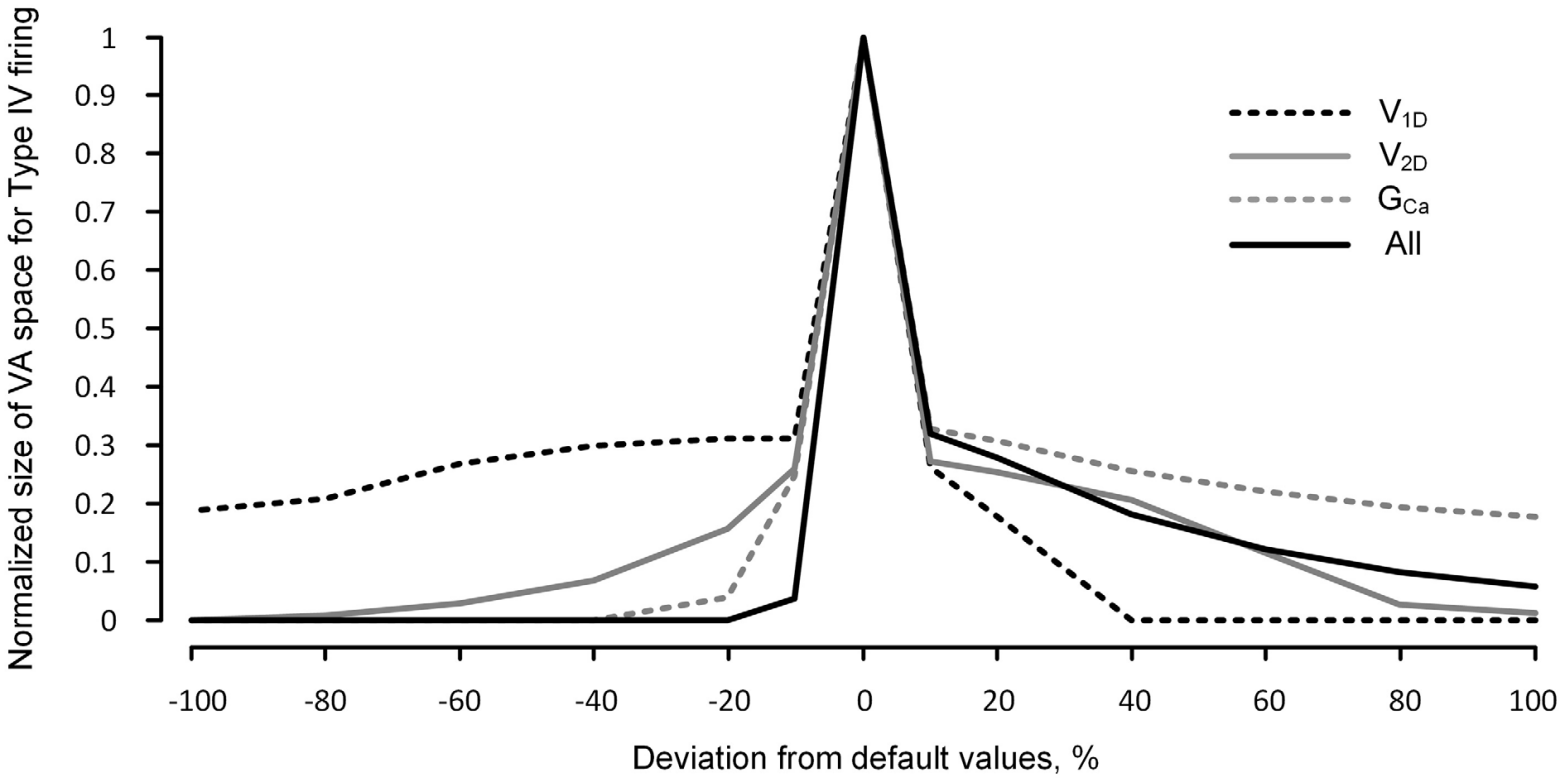
**Robustness of the reduced model for nonlinear firing under neuromodulation**. The same simulation for Figure 4 was performed for a broader range (from −100 to 100%) of change in the values of the individual and all activation parameters (*V_1D_*, *V_2D_*, and *G_Ca_*) of the CaPIC channel. The y-axis indicates the normalized number of the points in the 3D VA space where the reduced model produced the fully hysteretic Type IV firing (see the gray VA space in Figure 2 as an example with default values of the VA factors).

Figure 5 shows that the robustness of the reduced model for the Type IV firing is sharply reduced when the excitability of the dendrites is increased or decreased. In general, the reduced robustness of the model was more severe when the activation parameters varied in the inhibitory direction. Whether individual parameter values decreased or increased by 20%, the model robustness for Type IV firing decreased minimally by 70% in the case of *V_1D_* and maximally by 97% in the case of *G_Ca_*. When the activation parameter values were simultaneously changed by 10% in the same direction (such that all three parameters similarly increased or decreased dendritic excitability), the robustness was decreased by 97% in the inhibitory direction and 85% in the excitatory direction.

The dramatic reduction in the model's robustness for Type IV firing, in response to a relatively small change in the neuromodulation level, supports the idea that neuromodulation may control the firing mode of the motoneurons by modulating the influence of the PIC locations on the firing behavior.

## Discussion

Using a realistic two-compartment model, we theoretically investigated the neuromodulatory control of the firing behavior in motoneurons. The physiological firing patterns of the reduced model strongly related to the location of PIC channels in the dendrites. However, when the level of neuromodulation was either reduced or enhanced, the PIC locations estimated for the nonlinear (i.e., Type IV) firing behavior were almost abolished, and the whole solution space for Type IV firing in the 3D VA domain dramatically shrank. Consequently, neuromodulation could switch the reduced model between distinct firing modes (i.e., Type I and III), regardless of the PIC locations over the majority of the dendritic area. This result suggests that neuromodulation might play a role in controlling the firing mode of motoneurons by modulating the location dependency of PIC activation in the dendrites.

### Democratization of PIC impact on firing behavior through neuromodulation

The concept of democracy in the dendrites has been suggested both theoretically (Rumsey and Abbott, 2006) and experimentally (Magee and Cook, 2000; Hausser, 2001). That is, the contribution of individual synaptic inputs at different dendritic sites to the postsynaptic potentials at the soma could be normalized (or equalized), not only by the structure of the dendrites but also by passive and active membrane properties. A similar democratic phenomenon was found during the current study regarding PIC activation at different dendritic sites. In this study, the synaptic input and active current (i.e., PIC) in the dendrites were considered as extrinsic and intrinsic signals that may control the firing output at the soma. Similar to the synaptic input case, the firing output was investigated under neuromodulation while varying the location of PIC along the path of the dendrites from the soma. For this investigation, we used our recently developed reduced modeling approach for two reasons: (1) our reduced modeling approach provides the framework where the dendritic compartment can retain the dendritic excitability (i.e., input resistance) of the anatomically reconstructed models as a function of the distance by reflecting the voltage attenuation properties between the soma and the dendrites, and (2) the values for the cable parameters of the reduced model are analytically determined from the system properties (input resistance, time constant and three voltage attenuation factors) which is well suited for the generality of the simulation covering a full range of the voltage attenuation properties of the dendrites. Our simulation results showed that the effects of PIC channels' location on firing behavior could be normalized in a “democratic” manner under neuromodulatory control (Figure 4). For instance, when neuromodulation was reduced, the reduced model displayed only Type I firing without PIC activation, independent of the PIC location in the dendrites. However, when neuromodulation was enhanced, Type III firing with PIC activation at the initiation of firing was produced for PIC channels over most of the dendritic sites. This result suggests that neuromodulatory control might act as an extrinsic mechanism for democratizing the activation of the active channels over the dendritic trees.

### Robustness of type IV firing during normal behavior

Typically, when neuromodulation levels are fixed, the Type IV (fully hysteretic) firing pattern showing strong counter-clockwise hysteresis has been characterized to demonstrate the influence of PIC activation at the dendrites on firing behavior in spinal motoneurons. In the present study, the capability of the reduced motoneuron model to produce the Type IV firing was found to be highly sensitive to variations in the level of neuromodulation (Figure 5). That is, as neuromodulation increased, Type III firing became predominant for the PIC located over the majority of the dendritic area. This result might explain recent experimental observations both in animals and humans during normal behavior, which have demonstrated that the PICs tend to be activated almost simultaneously at the initiation of firing by synaptic inputs to the motoneurons, leading to a linear F-I relationship with sustained firing (i.e., Type III firing) (Gorassini et al., 2002a,b; Li et al., 2004). Taken together, these theoretical and experimental results suggest that the fully hysteretic, Type IV firing behavior might not be functionally important for normal movements, during which the neuromodulation level continuously varies in response to physical and emotional states.

### Limitations of the current modeling

The only firing type that was difficult to produce with this reduced model while varying the VA factors was Type II firing: a clockwise F-I relationship without sustained firing behavior (Figure 1). This result indicates that Type II firing does not seem to be related to the locations of the PIC in the dendrites. The underlying mechanisms for the firing adaptation during the falling phase of the triangular stimulation may be related to both passive and active membrane properties. A recent study of anesthetized hindlimb rat motoneurons has shown that the motoneurons with less input resistance tend to display the Type II pattern (Hamm et al., 2010; Turkin et al., 2010). Indeed, decreased input resistance in the reduced model led to the characteristics of Type II firing while blocking plateau-generating channels in the dendrite (not shown). For the active mechanisms, the slow kinetics of the voltage-gated Na^+^ and K^+^ (M-like) currents involved in shaping action potentials may be a factor contributing to the Type II firing pattern in mouse preparations (Iglesias et al., 2011). The K^+^ currents responsible for after hyperpolarization (AHP) may also affect the degree of the adaptation in the Type II firing of the reduced model. Both the input resistance and AHP property have been associated with motoneuron types (Zengel et al., 1985) and influenced by neuromodulation (Powers and Binder, 2001). Thus, further work will be needed to clarify if Type II firing is controlled by the neuromodulation in a type-specific manner in a pool of motoneurons. Other limitations to the modeling approach used in this study were addressed in detail in our previous studies (Kim and Jones, 2011, 2012). Briefly, the details of active currents involved in generating action potentials at the soma and plateau potentials at the dendrites have been collapsed into an inward and outward current at each compartment of the reduced model for the theoretical purpose of the present study. In addition, the current modeling approach may not be appropriate for the case where the PIC channels are located at multiple branches of the dendrites.

Detailed cellular mechanisms for the monoaminergic neuromodulation that facilitates PICs in spinal motoneurons are not yet clear (Heckman et al., 2009). The effects of monoamines on motoneuron excitability have been simulated by varying the peak conductance of K^+^ currents in the dendrites (Booth et al., 1997). In the current study, we varied three parameters (i.e., peak conductance, half activation voltage and slope of activation curve) that govern the activation of L-type Ca^2+^ channels in the dendrite of the reduced model to simulate neuromodulatory effects (Figure 3). We have found that both simulation approaches are comparable in that the excitability of the dendrite increases. However, what we found interesting in the current study was that changes in individual activation parameters had almost same effect on the PIC facilitation at different levels of monoamines. This result suggests that the monoamines might have compound effects on PIC activation in the dendrites, not only increasing the PIC amplitude but also varying the kinetic properties of the PIC. Furthermore, the activation properties of other voltage-gated inward and outward currents might also be varied under neuromodulation. Further systematic works would be needed to investigate whether or not and how the activation properties of non-PIC channels interact on the PIC location-dependent firing patterns under neuromodulation.

In the present study, individual firing types (i.e., Type I–IV) were identified based on temporal characteristics of the model response to a triangular current stimulation to the soma. Thus, one might concern that the firing types might depend on the kinetics of the triangular stimulation. With regard to this issue, we have shown in our previous studies (Kim and Jones, 2011, 2012) that Type I, III, and IV (fully hysteretic) firing could be defined mechanistically via steady-state bifurcation analysis. In addition, we could not find any significant difference in the firing types [Type I, III, and IV (fully hysteretic)] during simulations with a very slow (e.g., 10 time less steep) ramp stimulation (not shown).

Our intention of introducing the fully and partially hysteretic Type IV firing was to show the ability of the model to generate the two types of Type IV firing that have been experimentally observed in motoneurons. The main difference in the partially compared to fully hysteretic Type IV firings is the phenomenon of gradually deactivating plateau potential after the dendritic PICs are activated. In contrast to the fully hysteretic Type IV firing, the partially hysteretic Type IV firing was found to be sensitive to the kinetics of the ramp stimulation. Due to this reason, we have focused on Type I, III, and IV (fully hysteretic) firing for the theoretical purpose of the present study.

### Functional role of neuromodulatory control

Functional implications of the Type IV firing mediated by dendritic PICs have been suggested, not only for normal (i.e., gain and postural control) but also for pathological (i.e., spasticity) movement control (Kiehn and Eken, 1997; Lee and Heckman, 2000; Li and Bennett, 2003). Furthermore, it has also been reported that neuromodulatory control is likely to be predominant during physiological responses to external stimuli such as fight-or-flight-or-freeze behavior (Marder and Bucher, 2007; Inagaki et al., 2012; Suver et al., 2012). In all above cases, many motoneurons might need to fire together in a similar mode to ensure the strength and speed of muscle contraction required to generate those abrupt movements. This idea might be supported by our simulation results that the reduced models could be switched between distinct firing modes in a collective manner by varying the neuromodulation level, overriding the influence of PIC location on firing patterns (Figures 4, 5). Overall, neuromodulation might play a pivotal role in controlling the firing mode of motoneurons at the population level, instead of individually.

In conclusion, the monoaminergic inputs descending from brainstem nuclei to the motoneurons may differ depending on motor demands during normal behaviors. Variation of the neuromodulatory drive could adjust the influence of PIC location on the firing behavior in the reduced motoneuron models. Our simulation results suggest the hypothesis that neuromodulation may have a role in encoding the demanding motor states by switching the heterogeneous input-output properties of a population of motoneurons to a uniform operation mode.

## Author contributions

Conceived and designed the simulations: Hojeong Kim and C. J. Heckman. Performed the simulations: Hojeong Kim. Analyzed the data: Hojeong Kim and C. J. Heckman. Wrote the paper: Hojeong Kim and C. J. Heckman.

### Conflict of interest statement

The authors declare that the research was conducted in the absence of any commercial or financial relationships that could be construed as a potential conflict of interest.
